# Deep structural brain imaging via computational three-photon microscopy

**DOI:** 10.1117/1.JBO.30.4.046002

**Published:** 2025-03-29

**Authors:** Lingmei Chen, Mubin He, Lu Yang, Lingxi Zhou, Shuhao Qian, Chuncheng Wang, Rushan Jiang, Zhihua Ding, Jun Qian, Zhiyi Liu

**Affiliations:** a Zhejiang University, College of Optical Science and Engineering, International Research Center for Advanced Photonics, State Key Laboratory of Extreme Photonics and Instrumentation, Hangzhou, China; b Zhejiang University, Jiaxing Research Institute, Intelligent Optics and Photonics Research Center, Jiaxing Key Laboratory of Photonic Sensing and Intelligent Imaging, Jiaxing, China

**Keywords:** brain imaging, cerebrovasculature, three-photon microscopy, deep learning, morphological feature

## Abstract

**Significance:**

High-resolution optical imaging at significant depths is challenging due to scattering, which impairs image quality in living matter with complex structures. We address the need for improved imaging techniques in deep tissues.

**Aim:**

We aim to develop a computational deep three-photon microscopy (3PM) method that enhances image quality without compromising acquisition speed, increasing excitation power, or adding extra optical components.

**Approach:**

We introduce a method called low-rank diffusion model (LRDM)-3PM, which utilizes customized aggregation-induced emission nanoprobes and self-supervised deep learning. This approach leverages superficial information from three-dimensional (3D) images to compensate for scattering and structured noise from the imaging system.

**Results:**

LRDM-3PM achieves a remarkable signal-to-background ratio above 100 even at depths of 1.5 mm, enabling the imaging of the hippocampus in live mouse brains. It integrates with a multiparametric analysis platform for resolving morpho-structural features of brain vasculature in a completely 3D manner, accurately recognizing distinct brain regions.

**Conclusions:**

LRDM-3PM demonstrates the potential for minimally invasive *in vivo* imaging and analysis, offering a significant advancement in the field of deep tissue imaging by maintaining high-resolution quality at unprecedented depths.

## 1 Introduction

Imaging of brain structure and function is critical for monitoring vascular pathologies and pathogenesis of brain diseases, including Alzheimer’s and Parkinson’s diseases.^1^^–^^3^ Optical imaging has emerged as an optimal choice due to its high spatial resolution, real-time performance, and non-ionizing radiation. However, the strong scattering properties of biological tissues hinder their ability to observe the vascular system at substantial depths.^4^ Multiphoton microscopy is currently the preferred method for brain imaging in small animals.^5^ Two-photon excitation microscopy is fundamentally limited to an effective penetration depth of ∼300 to 700  μm due to scattering and defocused fluorescence background.^6^ In this regard, three-photon microscopy (3PM) has shown tremendous potential for further reducing scattering and suppressing unwanted background fluorescence by utilizing longer wavelengths and higher-order nonlinear excitation, thereby extending the imaging depth.^7^^,^^8^ Up to date, 3PM has been employed to study the static morphology of brain vasculature and neurons,^7^^,^^9^ as well as for calcium imaging of neurons^10^ and astrocytes.^11^ However, strong scattering from brain tissues still severely limits high-contrast optical imaging, with signal-to-background ratio (SBR) greatly degrading at large depths, which affects subsequent morpho-structural characterizations for resolving pathological alterations.

As a promising strategy, computational approaches based on deep learning for image restoration^12^^,^^13^ have the potential to improve the low SBR of images typically acquired from deep tissues. Although increasingly applied to images obtained using confocal, light-sheet, or two-photon microscopy setups,^14^^,^^15^ these methods have not yet been adjusted and customized for noise sources typically encountered at 3PM. Moreover, due to the complex nature of noise originating from weak fluorescence detection, such as the cooccurrence of structured and random noise in 3PM images, it might be difficult to obtain high-contrast images by dealing with different noise types using a single approach.

Supervised learning methods such as denoising convolutional neural network (DnCNN)^16^ face challenges in the microscopy domain due to the difficulty of obtaining paired data, whereas strategies using synthetic noise often underperform in real-world scenarios due to the lack of generalization capability. Recently developed self-supervised artificial intelligence–based methods, such as Noise2Self,^17^ Neighbor2Neighbor,^18^ and Noise2Fast,^19^ address noise by sampling noise pairs from single images for self-training, redistributing energy based on statistical assumptions. For natural images with a lot of spatial high-frequency components, these methods effectively hide noise within the signal, improving visual quality. However, for images without too many background details, such as brain vasculature images, these methods tend to make the background intensity approach the mean of noise, resulting in a mottled background and failing to enhance contrast. Denoising diffusion probabilistic models (DDPMs),^20^ a generative model implemented through denoising steps, holds promise for improving the quality of sparse microscopy images by combining denoising and generative capabilities. Nevertheless, DDPM also relies on random noise assumptions, making it unsuitable for the complex noise patterns in weak-light imaging, and requires clean images as the ground truth during training, limiting its ability to achieve true self-supervision. To overcome these technical limitations, we introduced a low-rank diffusion model (LRDM)-3PM, a three-photon imaging enhancement pipeline designed for complex noise in weak fluorescence detection, leveraging prior information on image quality degradation in three-dimensional (3D) microscopy imaging to achieve deep reconstruction.

In this study, we advanced 3PM and image analysis methods to enable a better understanding of vascular biology deep into white matter and the hippocampus. Specifically, we developed a minimally invasive *in vivo* imaging and analysis framework for 3D voxel-wise characterization of mouse brain vasculature. Using a type of home-made bright aggregation-induced emission (AIE) nanoprobes with a large three-photon absorption cross-section and diffusion-based deep learning post-processing tools (LRDM), which removed both detector and random noise, an SBR above 100 even at depths up to 1.5 mm was achieved. Finally, we demonstrated the ability of our method in mapping vascular architecture in deep white matter and the hippocampus of live mice.

## 2 Results

### 2.1 Workflow of Deep Brain Imaging

**Fig. 1:**
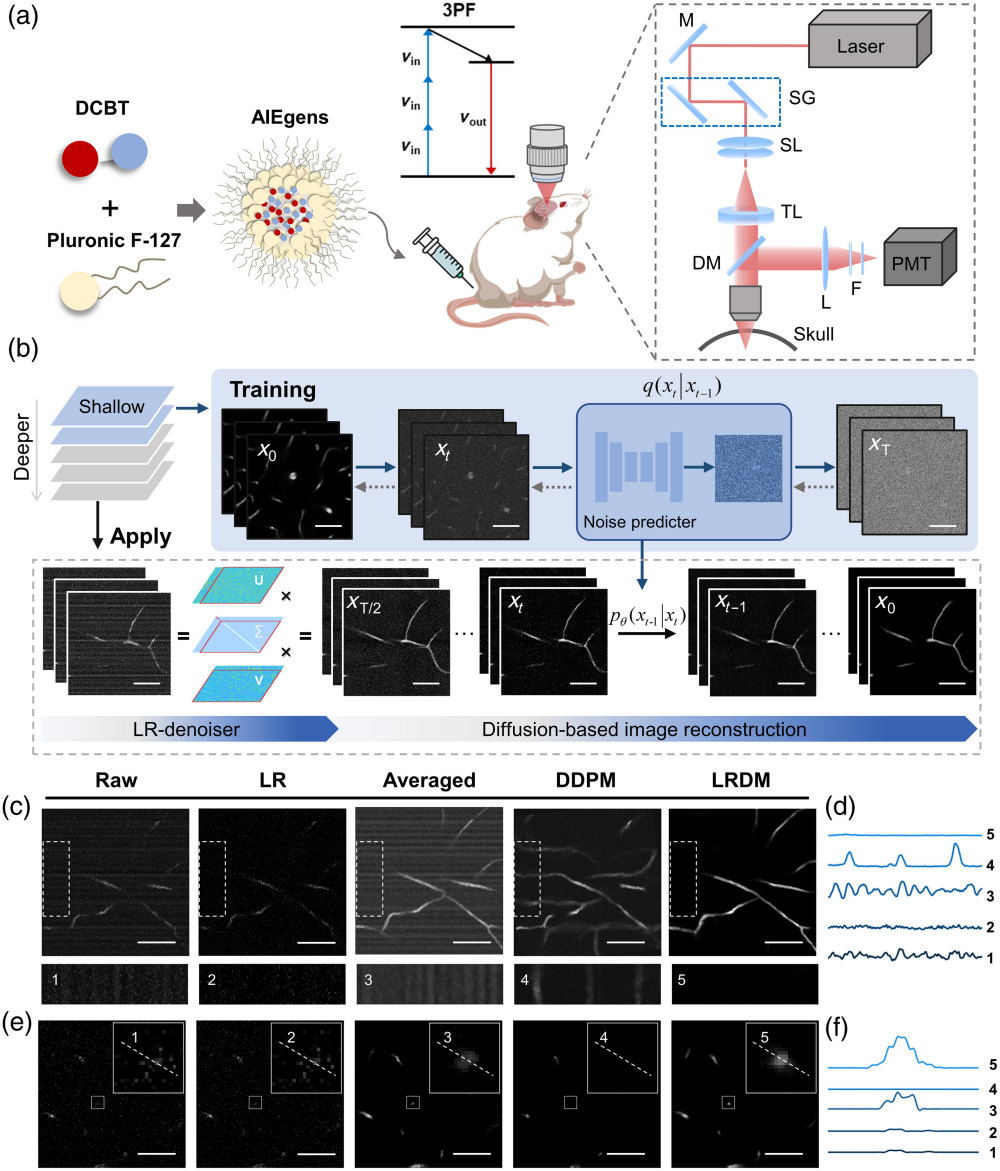
Pipeline for deep brain vessel imaging. (a) 3PM based on AIE nanoprobes. Illustrated are the design of the nanoprobe (left) and the optical path (right). M, mirror; SG, scanning galvanometer; SL, scan lens; TL, tube lens; DM, dichroic mirror; L, lens; F, filter. (b) Workflow of LRDM-3PM for denoising of raw images, including removal of structured noise from LR-denoiser and removal of random noise from a customized diffusion-based deep learning network. Scale bar, 50  μm. (c) Comparison of images following different processing strategies. Scale bar, 50  μm. (d) Intensity profiles along the vertical direction within the dashed box. (e) Convergence effect of the diffuse photon following different processing strategies. Scale bar, 50  μm. (f) Intensity profiles along the dashed line within the solid box.

To obtain three-photon fluorescence from mouse brain vasculature, we injected into the mouse tail a kind of bright AIE nanoprobes for efficient fluorescence collection [Fig. 1(a)].^21^^,^^22^ Specifically, an AIE fluorophore named (E)-2-(benzo[d]thiazol-2-yl)-3-(7-(diphenylamino)-9-ethyl-9H-carbazol-2-yl)acrylonitrile (DCBT) with a large three-photon absorption cross-section was made by intra- and inter-molecular synergistic engineering, and further encapsulated by F-127, which was approved by the US Food and Drug Administration, to form amphipathic organic nanoparticles (NPs). High-contrast images were obtained easily from the surface, but as depth increased, the image quality sharply deteriorated when light passed through the highly scattering region.^11^^,^^21^^,^^23^^,^^24^

To improve the quality of raw images while maintaining minimal 3PM excitation power to avoid photodamage, we proposed a deep learning–based denoising framework addressing both random noise and peculiar structured noise often encountered in deep 3PM images [Fig. 1(b) and Fig. S1 in the Supplementary Material]. Specifically, the scattering of light by biological tissues can be considered a diffusion process and can be simulated by certain diffusion models. Existing explicit modeling methods, such as Monte Carlo simulations, were complex and cumbersome, requiring substantial prior knowledge.^25^ On the other hand, some implicit modeling methods based on self-supervised deep learning^26^ failed to achieve optimal denoising effects due to the loss of signals in blind spots. In this regard, we developed a customized variant of the DDPM method^20^ for denoising deep 3PM data, referred to as LRDM-3PM. In our study, we utilized shallow data on 3D image sequences and performed data augmentation (Fig. S2 in the Supplementary Material) to train the diffusion model for image reconstruction. The forward process mimicked image degradation caused by scattering using the diffusion model, whereas the backward process employed a trained U-Net for noise prediction and removal to obtain clean images. To counteract the periodic structural noise patterns that the diffusion model could not address alone, we developed an additional preprocessing technique called low-rank (LR)-denoiser (Fig. S3 in the Supplementary Material) based on LR matrix decomposition theory (see Sec. 4), which preemptively removed the structured noise, thereby preventing the model from exacerbating it.

Notably, 3PM data contained a structured background such as ripple noise originating from the photomultiplier tube (PMT), which was modulated into a line-wise signal by the line scanning process. This type of noise violated the assumption of pixel independence, causing the established DDPM approach to either reproduce or amplify it and thus leading to errors in subsequent analysis. Besides structured noise, the random noise (mainly photon noise) also severely degraded the image quality, especially at large depths due to the lack of photons. Therefore, we modeled the obtained complex noise as a mixture of random and structured noise and performed targeted denoising aiming at both noise types via LRDM. Regarding structured noise, raw images contained stripes with similar intensity to the vascular signals [Fig. 1(c) and Fig. S4 in the Supplementary Material]; thus, the average step exacerbated the stripe appearance, whereas previous DDPM generated false vessels along the stripe direction. By contrast, as one of the key components of our LRDM method, the LR-denoiser effectively removed the stripe features [Fig. 1(d)]. Collectively, LRDM successfully removed both ripple noise from the detector and random noise from limited photons. Regarding the enhancement of signals [Fig. 1(e)], diffuse photons in the raw images were eliminated as noise by previous DDPM, unaffected by LR-denoiser, enhanced by averaging, and rendered most clearly using our approach [Fig. 1(f)]. Therefore, the LRDM framework effectively reduced background noise and enhanced signals, leading to improvement in contrast.

### 2.2 Performance and Validation of LRDM

#### 2.2.1 Performance of LRDM in improving the imaging quality

**Fig. 2:**
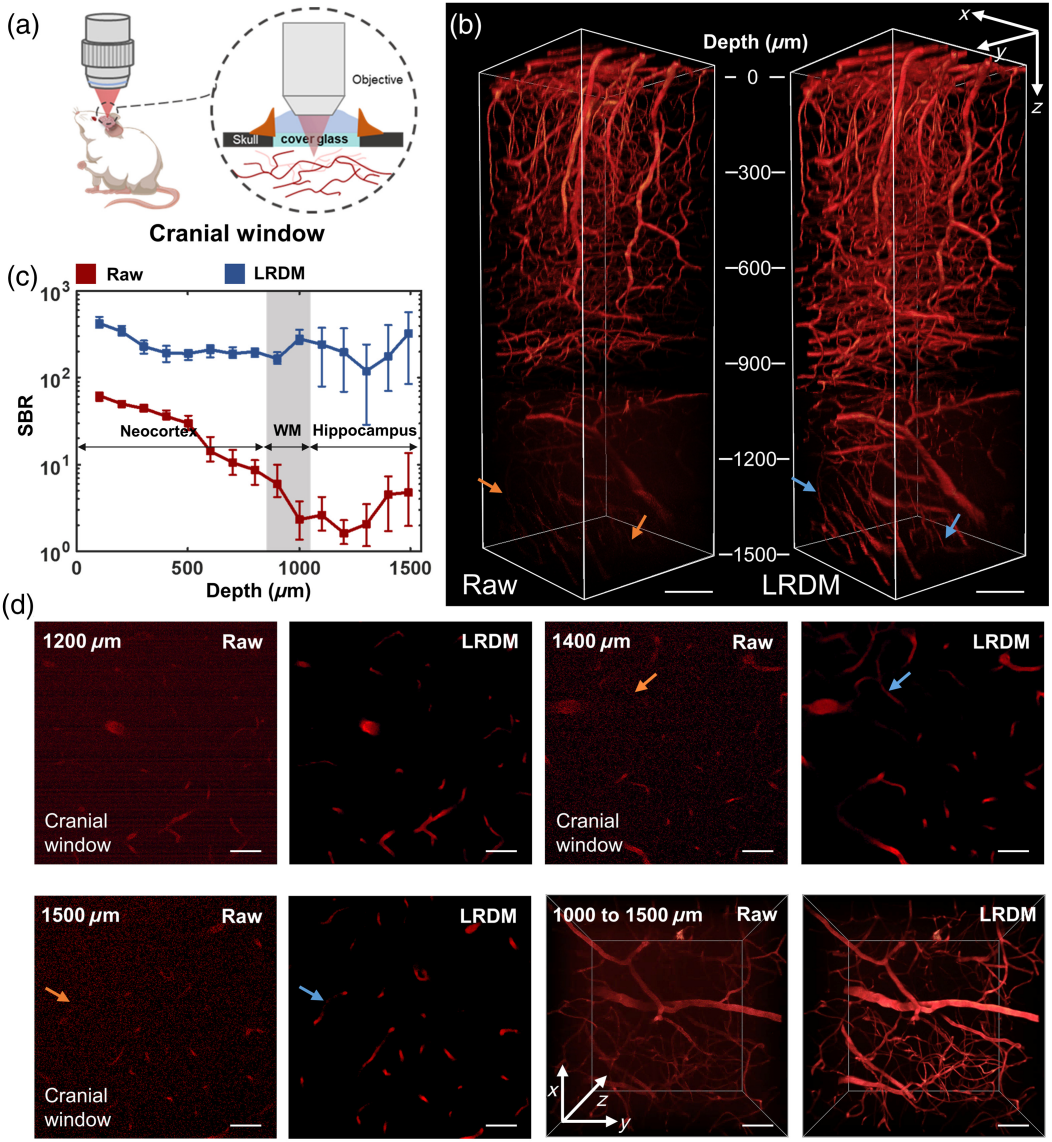
Performance of LRDM applied to the mouse cerebrovasculature with the cranial window. (a) Schematic showing mouse brain imaging with the cranial window. (b) 3D reconstructions of raw and LRDM-enhanced images. Arrows point to vessels that can be resolved from LRDM images but can hardly be identified from raw images. Scale bar, 100  μm. (c) SBR of raw and LRDM-enhanced images along depths. WM, white matter. (d) Comparison of raw and LRDM-enhanced images of mouse cerebral vessels at representative depths. Scale bar, 50  μm.

First, we applied LRDM to mouse cerebrovasculature obtained with the cranial window [Fig. 2(a)] to demonstrate the improvement in image quality achieved by this framework. For 3D reconstructions of images [Fig. 2(b)], vascular signals processed by LRDM between 900 and 1500  μm were visualized with high contrast, whereas for raw images, vessels beyond 900  μm were more difficult to identify, which might affect the subsequent segmentation and characterization.

To quantitatively assess the image quality before and after LRDM enhancement and to align the evaluation results directly with segmentation and quantification objectives, we adopted the SBR as the evaluation metric. Compared with some other metrics such as signal-to-noise ratio, SBR aimed to better distinguish between target and background signals, thereby providing a more reliable measure for downstream analysis tasks such as segmentation and characterization. From the quantitative comparison of raw and LRDM-enhanced images [Fig. 2(c)], we found that the SBR of raw images rapidly declined beyond the white matter region with the SBR falling below 10, whereas the enhanced images maintained a very high SBR level above 100, even reaching the hippocampus regions. Images from representative depth are shown in Fig. 2(d). As expected, image reconstruction especially at large depths was improved, making the faint deep vascular signals observable. As a result, consistent high-resolution vascular images throughout the entire 1500-μm depth were visualized.

**Fig. 3:**
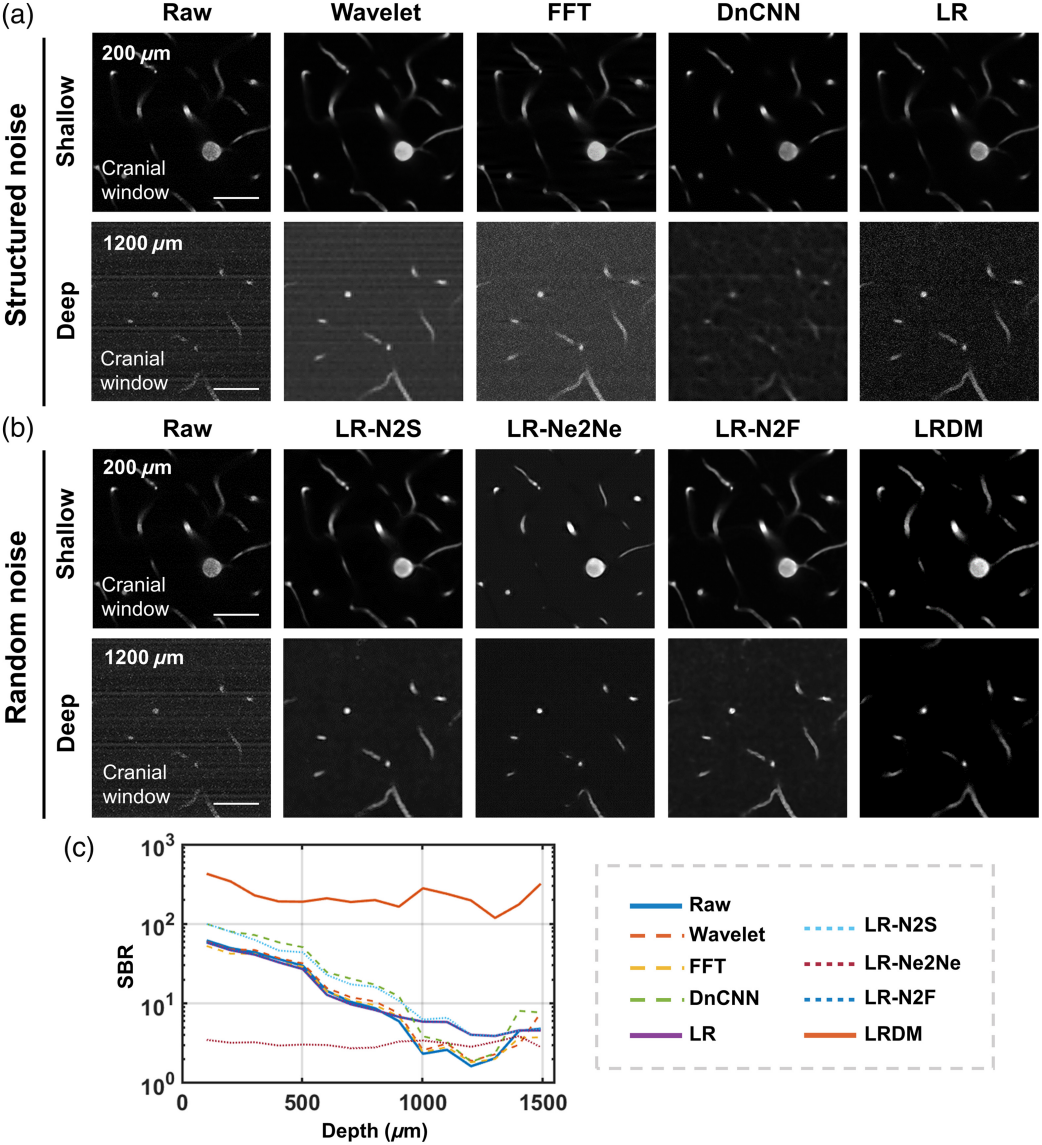
Evaluation of different denoising methods. (a) Comparison of different methods applied to structured noise. Wavelet, wavelet transform. FFT, frequency-domain bandpass filtering based on fast Fourier transform (FFT). Scale bar, 50  μm. (b) Comparison of different methods applied to random noise. N2S, Noise2Self. Ne2Ne, Neighbor2Neighbor. N2F, Noise2Fast. Scale bar, 50  μm. (c) SBR of images processed by different approaches along depths.

In addition, we compared the performance of different approaches for removing structured and random noise to demonstrate the superiority of our framework (Fig. 3). For structured noise [Fig. 3(a)], traditional methods such as wavelet transform and frequency-domain bandpass filtering exhibited limited capability in separating stripe noise and struggled to balance parameter selection, often introducing artifacts and distortions into images. Supervised models such as DnCNN required paired data for training, lacked generalization ability, and might be ineffective in addressing irregular stripe noise. By contrast, the images processed by the LR-denoiser exhibited noise that was closer to a random distribution, making them suitable for subsequent processing based on random noise assumptions. Following the LR-denoiser processing, for random noise [Fig. 3(b)], we compared our approach with several recent representative self-supervised learning methods (Noise2Self, Neighbor2Neighbor, and Noise2Fast). Analysis results revealed that these methods tended to “uniformize” the noise, whereas our LRDM method effectively “removed” it. We quantitatively evaluated the impact of these methods via SBR of mouse cerebrovascular images acquired through a cranial window [Fig. 3(c)]. Compared with other methods for removing structured noise, the LR-denoiser improved SBR in the range of 1000 to 1300  μm. LRDM consistently maintained a high level of SBR, whereas the combination of LR with other methods yielded SBR values nearly identical to those of LR alone, without obvious improvement.

#### 2.2.2 Validation of reconstruction reliability for LRDM

**Fig. 4:**
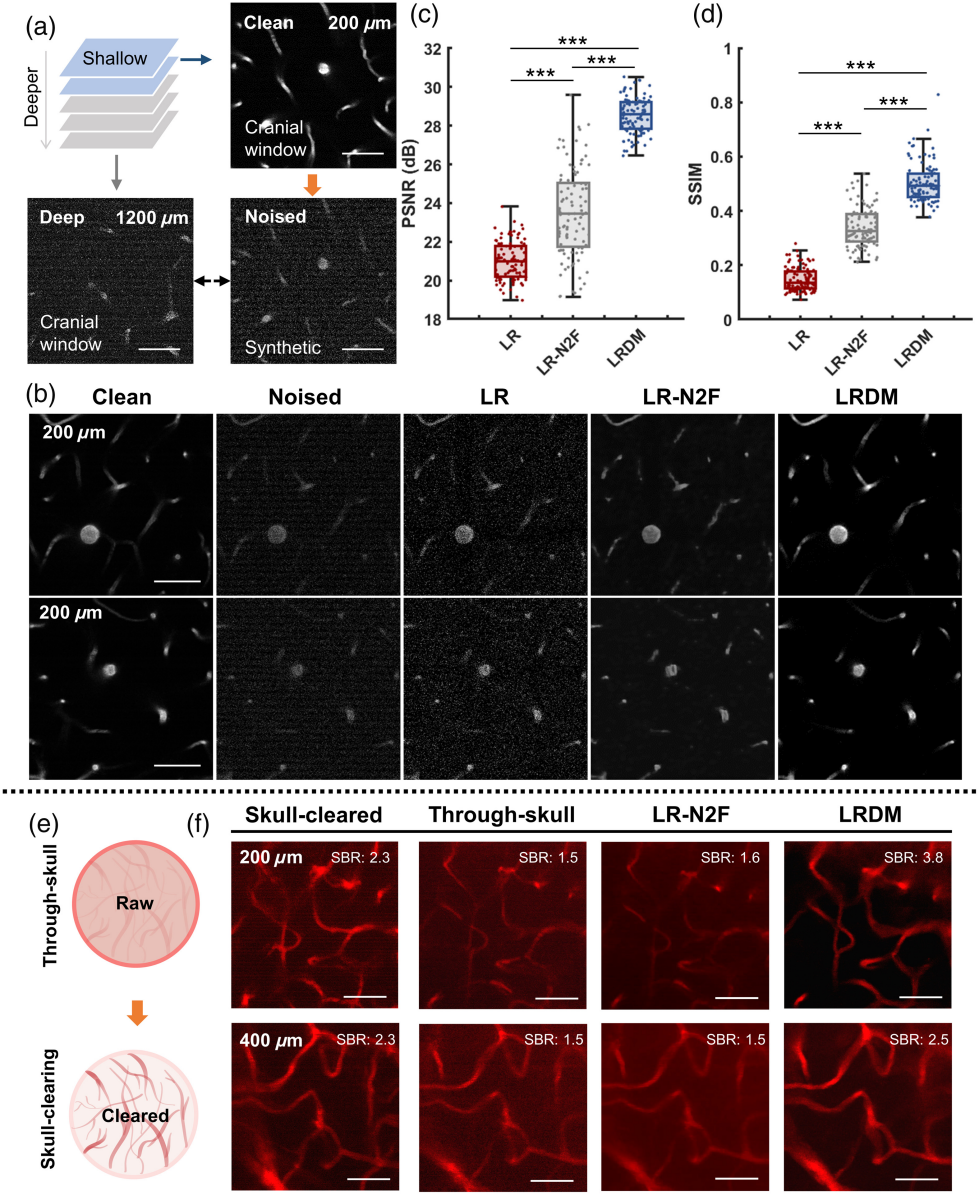
Validation of reconstruction fidelity for LRDM using paired data. (a)–(d) The process and results of paired validation using synthetic noise. (a) Schematic showing the generation of synthetic noised images by simulating deep images from shallow clean images. (b) Representative images corresponding to different denoising approaches. N2F, Noise2Fast. Scale bar: 50  μm. (c)–(d) Box plots of PSNR and SSIM values obtained from different approaches. n=100 images per group. ***p<0.001. (e)–(f) Paired data obtained via through-skull and skull-clearing methods. (e) Schematic showing the optical clearing of the skull. (f) Skull-cleared, through-skull, and enhanced (by LR-N2F and LRDM) images of the mouse brain vasculature at various depths, with SBR labeled in each image. N2F, Noise2Fast. Scale bar: 50  μm.

The authenticity of microscopic images is of paramount importance. To validate the reliability of the reconstruction results obtained by LRDM, we designed two sets of control experiments, encompassing tests on both synthetic data [Figs. 4(a)–4(d)] and real data [Figs. 4(e) and 4(f)]. First, we used images acquired from the cranial window at shallow depths as ground truth and added synthetic noise to mimic deeper imaging conditions [Fig. 4(a)]. We demonstrated the results obtained by processing the noised images using our proposed method and compared them with those from the latest self-supervised denoising methods [Fig. 4(b)], Noise2Fast^19^ (short as N2F). Again, to make a fair comparison, we applied LR-denoiser to raw images before treatment by N2F and named the whole process as LR-N2F. The analysis results revealed that the images reconstructed by LRDM were visually closer to the ground truth (clean images), which was further confirmed by quantitative image quality evaluation measures of peak signal-to-noise ratio [PSNR, Fig. 4(c)] and structural similarity index measure [SSIM, Fig. 4(d)]. We then designed a through-skull imaging experiment and compared the through-skull (without normalization) and enhanced vascular images with skull-cleared images, which were obtained following the treatment of the skull by a type of optical clearing agent (Fig. S5 in the Supplementary Material), termed visible-NIR-II compatible skull optical clearing agents (VNSOCA),^21^ to achieve high-contrast images of scattering brain tissues [Fig. 4(e)]. The LRDM-enhanced vascular images closely matched or even surpassed the contrast of cleared images, retaining clear vascular boundaries while suppressing diffuse background noise [Fig. 4(f) and Fig. S6 in the Supplementary Material].

**Fig. 5:**
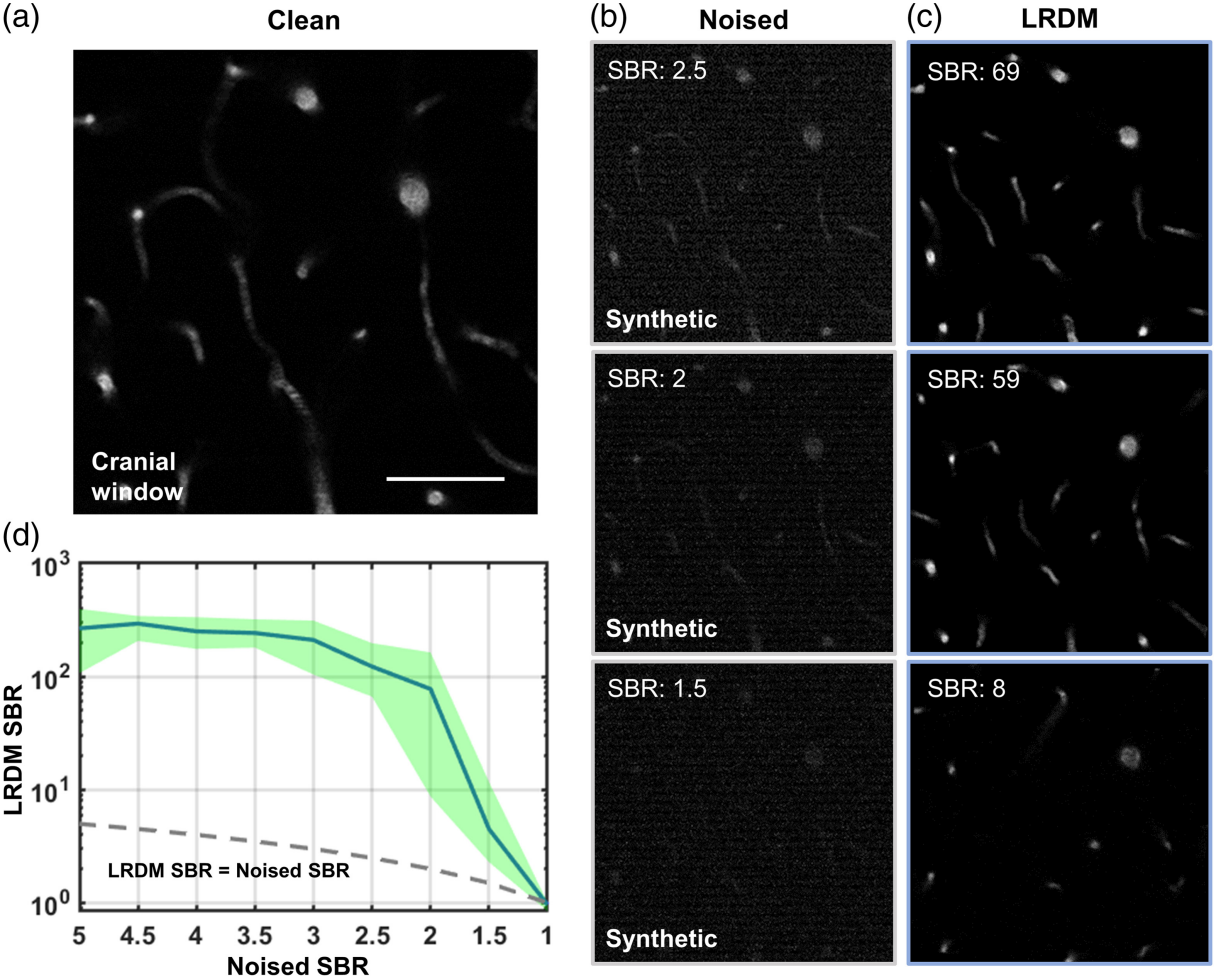
Relationship between noised and LRDM-enhanced SBR. (a) Shallow clean image shown as ground truth. Scale bar, 50  μm. (b) Synthetic noised images with different low SBR. (c) Corresponding LRDM reconstructions. (d) Plot showing the change of LRDM SBR as a function of noised SBR under low SBR conditions. The green shaded area represents the upper and lower limits of analysis results, n=10 images.

Furthermore, we performed simulations to explore LRDM’s reconstruction capability under low SBR conditions. By progressively degrading clean images [Fig. 5(a)], we found that LRDM achieved excellent reconstruction results when the SBR was >2. Even under extremely low SBR conditions, LRDM was still able to recover partially valid signals [Figs. 5(b) and 5(c), row 3]. The relationship between noised and LRDM-enhanced SBR was quantitatively demonstrated [Fig. 5(d)], further validating that a noised SBR of 2 might be the threshold.

### 2.3 Improvement in Vessel Segmentation

**Fig. 6:**
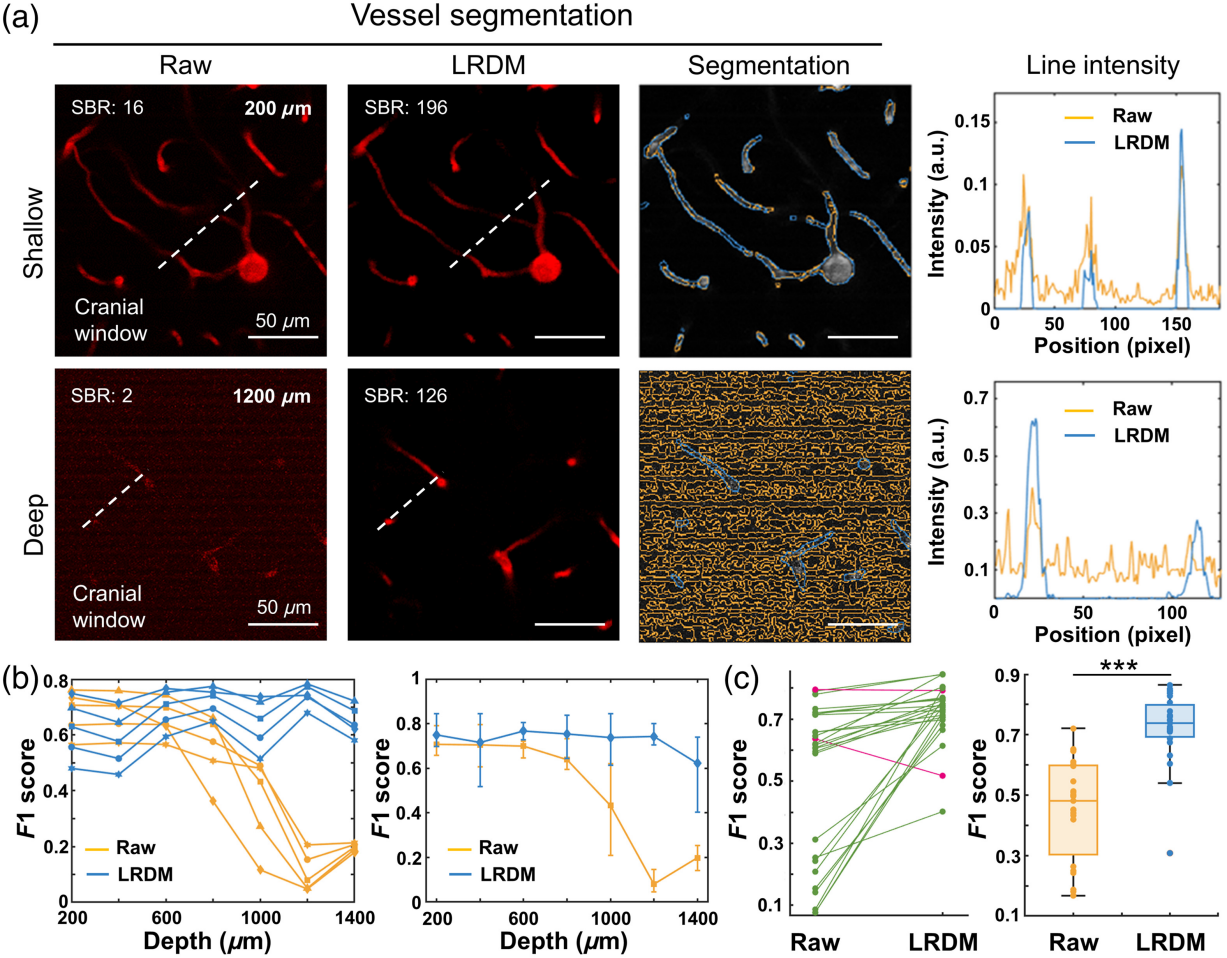
Improvement in vessel segmentation with LRDM. (a) Representative images and segmentation results at shallow and deep layers in response to LRDM enhancement (left), and intensity profiles along the white dashed lines (right). SBR values are marked in representative images. Scale bar, 50  μm. (b) The segmentation accuracy evaluated by the F1 score as a function of imaging depth, using the proportion-adjusted Otsu thresholding (left) and the weighted optimal thresholding (right). (c) Left, slope chart showing segmentation results at distinct 2D slices. Each line corresponds to one slice image, with increased accuracy colored by green, and decreased accuracy colored by magenta. n=27 images from four mice. Right, box plots showing segmentation accuracy considering the entire 3D stack. ***p<0.001.

Previous studies suggested that denoising was closely related to vessel segmentation.^19^^,^^27^ Thus, segmentation results based on raw and LRDM-enhanced images were compared. Here, the segmentation was done through automatic Otsu thresholding,^28^ and the accuracy was determined by comparing segmentation results with manually annotated ground truth. As can be seen, the enhanced images allowed for the segmentation of continuous vessels and maintained accurate outputs even at deep zones, where vessel classification could hardly be achieved from raw images suffering from severe striped and random noise [Fig. 6(a) and Fig. S7 in the Supplementary Material]. The intensity profiles to the right from both images highlighted the importance of improvement in image contrast [Fig. 6(a)]. As quantified from the F1 score, which was commonly used as an evaluation parameter in binary and multiclass classification, readouts from both the proportion-adjusted Otsu thresholding [Fig. 6(b), left] and the weighted optimal thresholding [Fig. 6(b), right] revealed that segmentation accuracy from enhanced images remained stable with increasing depth and was much higher than that from raw images, especially at deep zones (Fig. S8 in the Supplementary Material). Considering segmentation results at all different depths, the slope chart [Fig. 6(c), left] illustrated that the segmentation of most two-dimensional (2D) slices improved (green line) following enhancement, and a significant difference was obtained when assessing the segmentation through the entire 3D stack [Fig. 6(c), right].

### 2.4 Multiparametric Characterization of Mouse Brain Vessel Based on Accurate Segmentation

**Fig. 7:**
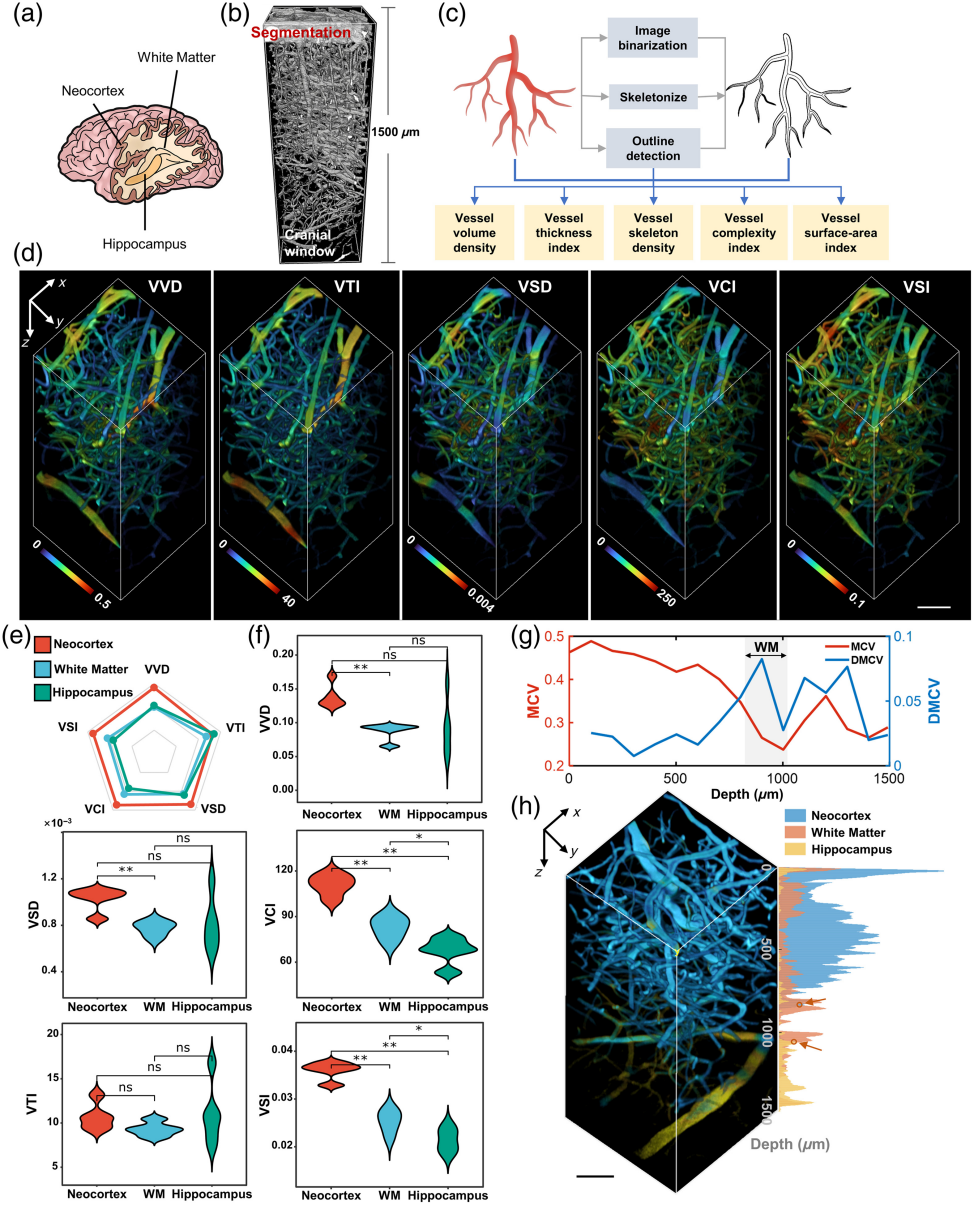
3D vessel quantitation based on accurate segmentation. (a) Illustration of mouse brain regions. (b) Vessel segmentation through the 1.5-mm depth in mouse brain from the LRDM-enhanced image. (c) Flowchart of the 3D multiparametric analysis. (d) 3D reconstructions of color-coded maps of five 3D optical metrics describing morpho-structural features of cerebrovasculature. Scale bar, 50  μm. (e) Radar chart corresponding to the average level of normalized morpho-structural metrics. (f) Violin plots showing statistical analysis results for each metric. n=4 specimens for each brain region. *p<0.05, **p<0.01. ns, nonsignificant. (g) Line charts showing MCV and absolute DMCVs for each metric. WM, white matter. (h) White matter recognition based on vascular morphology. Scale bar, 50  μm (Video 1, MP4, 10.2 MB [URL: https://doi.org/10.1117/1.JBO.30.4.046002.s1]).

Accurate segmentation is fundamental to morpho-structural characterization of vasculature. Empowered by LRDM-3PM, we were able to achieve accurate vessel segmentation across three brain regions [Fig. 7(a)] over 1500  μm [Fig. 7(b)], which paved the way for the following quantitative vessel characterization down to the mouse hippocampus. To guarantee architectural fidelity, we extended the classic 2D vascular morphological parameters commonly used for evaluating vascular projection images^29^ to voxel-wise 3D optical metrics [Fig. 7(c), see Sec. 4], including vessel volume density (VVD), vessel thickness index (VTI), vessel skeleton density (VSD), vessel complexity index (VCI), and vessel surface-area index (VSI). Owing to the voxel-wise nature of these metrics, we obtained color-coded heat maps for direct visualization and demonstration [Fig. 7(d), Video 1], enabling the analysis of differences in vascular morpho-structural features across distinct brain regions.

We obtained depth-dependent profiles and corresponding distribution histograms of each metric (Fig. S9 in the Supplementary Material). Specifically, we observed the presence of large vessels from the white matter layer to the hippocampal layer [Fig. S9(b) in the Supplementary Material], consistent with previous results from 3PM deep imaging studies.^11^^,^^23^^,^^24^ Within the hippocampal region, the vessel thickness exhibited substantial variation, especially shown by VVD and VTI charts [Fig. S9(b) in the Supplementary Material]. In addition, a second peak originating from large vessels appeared in distribution histograms [Fig. S9(c) in the Supplementary Material, arrows]. With increasing depth, an overall decreasing trend was observed for all parameters, and VSD, VCI, and VSI exhibited a transient increase between 0 and 500  μm due to vascular differentiation, consistent with previous findings.^30^^,^^31^ These distributions implied that the main components across different brain regions were small vessels with similar diameters, whereas the vascular distribution and morphology were different among distinct brain regions.

We then compared the mean values of these vascular metrics for the neocortex, white matter, and hippocampus. Following normalization, results were presented in a radar chart [Fig. 7(e)]. As can be seen, VTI exhibited a different relationship among brain regions compared with other metrics, attributed to the presence of large blood vessels in the hippocampus. We then obtained violin plots for each metric [Fig. 7(f)]. Generally, these results were in accordance with those shown from depth-dependent profiles and distribution histograms. Especially, we found that both VCI and VSI were sensitive measures in identifying distinct architectural features among brain regions.

The coefficient of variation (CV) was used to represent the dispersion of vascular metrics. As observed from the CV-depth line chart for each metric (Fig. S10 in the Supplementary Material), similar trends were acquired, with a higher level in the neocortex than that in white matter and hippocampus. We then acquired the mean CV (MCV) and the absolute difference of MCV (DMCV) for a more intuitive display [Fig. 7(g)]. It was evident that vascular heterogeneity underwent a substantial change near white matter by reaching a minimum and then increased again deep into the hippocampus. The majority of these metrics were independent, thus offering complementary insights by satisfying the absolute correlation coefficient below 0.7, as suggested from the correlation heatmap among vascular metrics (Fig. S11 in the Supplementary Material).

Following the acquisition of region-specific vascular features, we employed a support vector machine (SVM) method to construct an automatic model for brain region identification. Using features extracted from 3PM images of three mice, we trained a linear SVM model and validated its accuracy using an independent validation dataset [Fig. S12(a) in the Supplementary Material]. Analysis results revealed that the voxel-wise accuracy combining all metrics (group 5) was close to 85%, higher than other representative metric combinations [Fig. S12(b) in the Supplementary Material]. In the depth-dependent distributions of voxel-wise classification results, we identified crossover points where the counts of white matter voxels surpassed that of the neocortex and hippocampus [Fig. 7(h), arrows] located at 837 and 1057  μm, respectively, which enabled estimation of the depth belonging to the white matter region. The measured depth of white matter by three-harmonic generation (THG) imaging^21^ was ∼850 to 1050  μm, highly consistent with our observations.

## 3 Discussion and Conclusion

In this study, we develop an *in vivo* imaging framework termed LRDM-3PM. Specifically, we employ AIE nanoprobes-based 3PM for large-depth cerebrovasculature imaging and further combine it with artificial intelligence–driven image restoration, enabling the maintenance of an unprecedented SBR above 100 even at a depth of 1.5 mm in live mouse brains. High-contrast vessel imaging reaching the mouse hippocampus paves the way for studying alterations in vascular morphology, which is highly relevant to brain pathophysiology.^11^^,^^26^

The denoising technique developed in this study, LRDM, integrates LR matrix decomposition theory and probabilistic diffusion models to achieve stable and effective noise suppression and signal compensation, thereby improving the contrast of fluorescence microscopy. Specifically, the structured background is effectively removed by the LR decomposition, which was previously validated to be powerful in dealing with such background because the relatively sparse distribution of stripes exhibited a latent LR structure.^32^ It is noteworthy that our method is entirely based on linear algebra and mathematical statistics, providing a solid theoretical foundation. Although achieving superior performance, it offers high interpretability, well adapting and aligning with the stringent requirements for image rigor in biomedical applications.^33^ As validated experimentally, LRDM operates well in a self-supervised way, circumventing the need for training with hundreds of thousands of high-quality images, which might be difficult to obtain, especially 3D data.^34^^,^^35^ LRDM can compensate for sample scattering and pattern noise without recourse to adaptive optics (AO) and high excitation power, which would be useful for most labs lacking access to sophisticated AO setups while intending to improve the image quality acquired from existing hardware. We perform a series of experiments to make a detailed comparison of the superior performance of LRDM with established methods and demonstrate its ability to alleviate the rapid decrease in SBR deep within tissues. Furthermore, we illustrate that LRDM achieves reliable reconstruction even under extremely low SBR conditions (Fig. 5). The SBR threshold value could potentially indicate the depth limit of imaging, although it is not obtained experimentally in this study and will be our future work.

Empowered by the exceptional noise suppression and signal enhancement from LRDM-3PM, the vessel segmentation especially beyond 1 mm is greatly improved, which enables the acquisition of quantitative architectural information of vasculature across three brain regions. In this regard, we develop a multiparametric 3D analysis platform that integrates a series of morpho-structural features, including volume and skeleton density, thickness, complexity, and surface area of blood vessels, in a completely 3D manner, which is proved to have a higher sensitivity than its 2D counterpart.^36^^,^^37^ We observe depth-dependent trends in the density and morphology of vascular networks and find that these morpho-structural features are significantly different across distinct brain regions. These metrics are tested to be basically independent of each other, thus offering complementary insights from different angles and providing new references for understanding the relationship between region-associated functional roles and vascular network architecture. Building upon this, our SVM-based classification model constructed using these developed 3D vascular features has demonstrated the potential to identify different brain regions. The estimated white matter scale closely matches the identification from THG imaging, avoiding the need for secondary sample imaging and system transitions and, more importantly, offering potential with promise for recognizing structural changes in cerebrovasculature induced by brain diseases.^2^

In summary, LRDM-3PM enables progress in deep tissue imaging, providing a powerful tool for the acquisition of high-contrast deep brain vessel images and quantitative analysis. This technique compensates for scattering from deep tissues, improving imaging quality without requiring any external data or system components. The exceptional imaging performance achieved by this method enhances the segmentation of vessels, which is fundamental to downstream analysis. Integrating the deep imaging with the multiparametric analysis, we have extended the automated voxel-wise vascular analysis to unprecedented depths, especially in a truly 3D context that has not been reported previously, and revealed differences in vascular morphology across distinct brain functional regions. As suggested by the literature that neurodegenerative diseases might be related to alterations in vascular morphology,^1^^–^^3^ our method provides a unique perspective for understanding the mechanisms and diagnosis of brain disorders and might potentially offer insights for the development of new therapeutic strategies.

## 4 Materials and Methods

### 4.1 Brain Vasculature Imaging

#### 4.1.1 Preparation of DCBT NPs

DCBT NPs were prepared according to the standard procedure reported previously.^38^ Briefly, 1-mg DCBT molecules and 12-mg Pluronic F-127, all dissolved in 0.5-mL tetrahydrofuran (THF), were mixed together and sonicated for 9 min. Then, the mixture solution was dropped into 12-mL deionized water. Afterward, the residue THF was evaporated in the fume hood by stirring for 5 h. Finally, the DCBT NP solution was concentrated in an ultrafiltration tube.

#### 4.1.2 3PM system

3PM system included two major parts, a noncollinear optical parametric amplifier (NOPA) with wavelength-tunable femtosecond (fs) laser output and a commercial Bruker scanning microscope. The NOPA system included a 1030-nm fs pump laser (Spectra-Physics, Spirit, Milpitas, California, United States) and an OPA system (Spectra-Physics, NOPA-VISIR). A 1300-nm fs laser beam (115 fs, 1 MHz) was introduced into the scanning microscope as the excitation source. The excitation beam was focused on the sample through an objective (XLPLN25XWMP2, Olympus, Center Valley, Pennsylvania, United States, NA = 1.05), and the excited three-photon fluorescence was then collected by a GaAs PMT (H7422–40, Hamamatsu), after reflected by a dichroic mirror (Semrock, DMLP 700, Rochester, New York, United States) and passing through the filter (Semrock, FF02–641/75).

#### 4.1.3 *In vivo* 3PM cerebrovascular imaging in mouse

The C57 mouse (male, 8 to 10 weeks old) was anesthetized by pentobarbital sodium (0.14 mL, mass concentration = 1%), and a cranial window with a diameter around 6 mm was produced by removing the scalp and a small piece of skull. After being injected with DCBT NPs (1.5  mg/mL, 200  μL) via the tail vein, the mouse was immobilized on a lab-built plate and imaged under the 3PM system. The z-stack images were taken at 2-μm step, and the scanning speed was 2.2  μs/pixel. There were four mice used for skull-free imaging. For skull-clearing imaging, which was used to validate LRDM performance, we used a type of home-made optical clearing agent, named VNSOCA, for the treatment of mouse skull, detailed in our previous work.^21^^,^^39^ All animal experiments performed in this study were conducted strictly in compliance with the ethical standards of the Institutional Ethical Committee of Animal Experimentation of Zhejiang University.

### 4.2 LRDM-3PM Deep Learning–Based Denoising

#### 4.2.1 LR-denoiser

To eliminate the periodic structural noise characteristic of 3PM, we constructed a dedicated pre-denoising model called LR-denoiser (Fig. S3 in the Supplementary Material). This approach employed LR matrix decomposition theory to remove the periodic structural noise in 3PM. Initially, the singular value decomposition (SVD) method was used to achieve the orthogonal decomposition results of the image. As for an input image of size M×N (M≥N), it was possible to represent this image in the r-dimensional subspace, where r is the rank of X, with r≤N

$$X={\mathrm{USV}}^{T},$$ (1)

where U is an M×r matrix consisting of orthonormalized eigenvectors of $XX^{T}$ and V is an N×r matrix consisting of orthonormalized eigenvectors of $X^{T}X$. S is an r×r diagonal matrix consisting of singular values of X, which are the nonnegative square roots of eigenvalues of $X^{T}X$. These singular values, denoted by σ, were sorted in non-increasing order, i.e., $\sigma_{1}\geq \sigma_{2}\geq \cdots \geq \sigma_{r}\geq 0$.

For images severely affected by stripe noise, only a few larger singular values dominated, significantly higher than all other singular values. By contrast, the singular values of normal images had a more uniform distribution. Therefore, we replaced the larger singular value to impair the periodic structural noise of the image and removed such background texture by reconstructing the image with the new singular values.

Subsequently, an image reconstruction based on SVD was performed. By calculating the ratio of the largest singular value to the second one, we filtered out the noise images that met the threshold criterion. In our study, the threshold ratio was set to 3. The image reconstructed from the new singular values was given by

X^=∑j=1rUjσjVjT, (2)

where $\hat{X}$ is the reconstructed image, $U_{j}$ and $V_{j}$ are j’th column vectors of U and V, respectively, $\sigma_{j}$ is j’th singular value of S, and r is the rank of the matrix X.

#### 4.2.2 Network architecture

In our approach, we designed a customized version of the classic U-Net architecture^40^ [Fig. S1(b) in the Supplementary Material]. Our model used four feature map resolutions (256×256 to 32×32). This model had two convolutional residual blocks per resolution level and self-attention blocks at the 64×64 resolution between the convolutional blocks. Diffusion time t was specified by adding the Transformer sinusoidal position embedding^41^ into each residual block.

#### 4.2.3 Customized DDPM

First, we extracted subvolumes from the shallow side of the experimentally acquired image stacks, using these data as ground truth [Fig. S1(a) in the Supplementary Material]. We defined the “shallow side” of an image stack by the planes nearest to the detection objective, which were typically performed with the best image quality. Second, those ground truth images were used to train the diffusion model by adding and removing Gaussian noise step by step. In our work, we set the diffusion process length T=200 in the training procedure and used the adaptive moment estimation (ADAM) optimizer^42^ to minimize the loss value over 300 epochs. During the inference procedure, all the images were dealt with by the LR-denoiser to remove the stripe noise and then averaged by the intensity of their nearby depth. After these pre-processing steps, we applied the trained model to reduce scattering noise in experimentally acquired image volumes.

#### 4.2.4 Evaluation of denoising performance

To assess changes in imaging contrast before and after LRDM enhancement, we employed the SBR as the evaluation metric, calculated as follows:

$$\mathrm{SBR}=\frac{\mu_{\text{Signal}}}{\mu_{\text{Background}}},$$ (3)

where $\mu_{\text{Signal}}$ represents the average intensity of the target signal and $\mu_{\text{Background}}$ represents the average intensity of the background. In our study, the target regions were manually annotated. A higher SBR revealed a higher contrast in the image.

Two types of metrics were used for quantitative validation of the reconstruction reliability of LRDM. For the evaluation of image quality, we obtained ground truth images with high contrast by skull optical clearing and compared the SSIM of images before and after enhancement. SSIM was calculated as

$$\mathrm{SSIM}(u,v)=\frac{\left(2\mu_{u}\mu_{v}+Q_{1})(2\sigma_{uv}+Q_{2}\right)}{\left(\mu_{u}^{2}+\mu_{v}^{2}+Q_{1})(\sigma_{u}^{2}+\sigma_{v}^{2}+Q_{2}\right)},$$ (4)

where $\mu_{u}$ and $\mu_{v}$ are average gray values, $\sigma_{u}$ and $\sigma_{v}$ are variance of patches, $\sigma_{uv}$ is the covariance of u and v, and $Q_{1}$ and $Q_{2}$ denote two small positive constants (typically 0.01).

As another evaluation metric, PSNR was determined through the mean square error (MSE), which was the average of the square of the difference between the original image and the denoised image, defined as

$$\mathrm{MSE}=\frac{1}{N}{\left\|I-L\right\|}^{2},$$ (5)

where I is the gray values of ground truth, L represents the test image, and N is the number of pixels. Lower MSE values signified better image quality. Then, PSNR was an engineering term that measured the ratio between the maximum original signal and MSE, with a higher PSNR value representing better image quality. PSNR was defined as

$$\mathrm{PSNR}=10^{*}\log_{10}(\frac{(\max(I){)}^{2}}{MSE}).$$ (6)

Furthermore, we evaluated the performance on the downstream task such as vessel extraction. We considered vessel extraction as an instance segmentation problem and adopted an object-level metric to evaluate the segmentation results of the Otsu method before and after denoising. The segmentation accuracy (F1 score) was defined as the harmonic mean of sensitivity and precision, formulated as

$$F_{1}=\frac{2\times \mathrm{TP}}{2\times \mathrm{TP}+\mathrm{FP}+\mathrm{FN}},$$ (7)

where TP, FP, and FN are the number of true positives, false positives, and false negatives, respectively.

### 4.3 Morpho-Structural Characterization of Brain Vessels

#### 4.3.1 3D multiparametric analysis model

Here, several widely accepted statistical parameters were calculated and expanded to 3D voxel-wise ones, including VVD, VTI, VSD, VCI, and VSI, with the definitions given as

$$\mathrm{VVD}=\frac{\overset{n}{\underset{x=1}{\sum }}\overset{n}{\underset{y=1}{\sum }}\overset{n}{\underset{z=1}{\sum }}A(x,y,z)}{n^{3}},$$ (8)

$$\mathrm{VTI}=\frac{\sum_{x=1}^{n}\sum_{y=1}^{n}\sum_{z=1}^{n}A(x,y,z)}{\sum_{x=1}^{n}\sum_{y=1}^{n}\sum_{z=1}^{n}S(x,y,z)},$$ (9)

$$\mathrm{VSD}=\frac{\sum_{x=1}^{n}\sum_{y=1}^{n}\sum_{z=1}^{n}S(x,y,z)}{n^{3}},$$ (10)

$$\mathrm{VCI}=\frac{[\overset{n}{\underset{x=1}{\sum }}\overset{n}{\underset{y=1}{\sum }}\overset{n}{\underset{z=1}{\sum }}P(x,y,z){]}^{2}}{4\pi \overset{n}{\underset{x=1}{\sum }}\overset{n}{\underset{y=1}{\sum }}\overset{n}{\underset{z=1}{\sum }}A(x,y,z)},$$ (11)

$$\mathrm{VSI}=\frac{\sum_{x=1}^{n}\sum_{y=1}^{n}\sum_{z=1}^{n}P(x,y,z)}{n^{3}},$$ (12)

where A(x,y,z) represents voxels registered as vessel volume, S(x,y,z) represents voxels registered as vessel skeleton, and P(x,y,z) represents voxels registered as vessel surface. The superscript n refers to the width, height, and depth of the target image. VVD represented the number of vessel voxels per unit volume. VTI indicated the diameter of vessels in 3D space. VSD represented the number of vascular skeleton voxels per unit volume, reflecting the density of the vascular network. VCI was a concept derived from the field of image processing, aiming to describe the irregularity of vascular morphology. Finally, VSI denoted the number of voxels representing the vascular surface area per unit volume, reflecting the efficiency of blood flow exchange.

#### 4.3.2 Machine learning–based classification of brain regions

The classification model proposed in this study was constructed based on the training results of the SVM method. After VVD, VTI, VSD, VCI, and VSI in each region were obtained, termed $a_{i}$, $b_{i}$, $c_{i}$, $d_{i}$, and $e_{i}$, respectively, vector ${\overset{\to }{V}}_{i}=[a_{i},b_{i},c_{i},d_{i},e_{i}]$ and label vector ${\overset{\to }{L}}_{i}$ ($L_{i}=1$ corresponded to the neocortex, $L_{i}=2$ corresponded to the white matter and $L_{i}=3$ corresponded to the hippocampus) were generated and trained by the SVM to determine the decision surface

$$k^{T}\overset{\to }{V_{i}}+b=0.$$ (13)

As the normal vector $\overset{\to }{k}$ and intercept b were obtained, the classification of the sample was generated by

$$\text{results}(x,y,z)=\overset{\to }{k}\cdot \overset{\to }{V}(x,y,z)+b,$$ (14)

where $\overset{\to }{V}(x,y,z)=[a(x,y,z),b(x,y,z),c(x,y,z),d(x,y,z),e(x,y,z)]$. Here, a(x,y,z),b(x,y,z),c(x,y,z),d(x,y,z),e(x,y,z) represents the voxel-wise quantification results with (x,y,z) representing the position on the map.

#### 4.3.3 Statistical analysis

For a quantitative comparison of segmentation accuracy with raw and LRDM images, a student’s t-test was performed. A one-way analysis of variance (ANOVA) post-hoc Tukey honestly significant difference (HSD) test was performed to assess differences among neocortex, white matter, and hippocampus brain regions. Differences were considered statistically significant at p<0.05.

## Supplementary Material

10.1117/1.JBO.30.4.046002.s01

10.1117/1.JBO.30.4.046002.s1

## Data Availability

Data and code developed in this study are available upon reasonable request to the corresponding author.
