# Supplementary material for: Deep structural brain imaging via computational three-photon microscopy
Source: J Biomed Opt. 2025 Mar 29;30(4):046002. doi: 10.1117/1.JBO.30.4.046002 (PMC11954598; doi:10.1117/1.JBO.30.4.046002)
Supplement: Supplementary file 1 [file JBO_030_046002_SD001.pdf]

Deep structural brain imaging via computational three-photon microscopy

Supplementary Figures

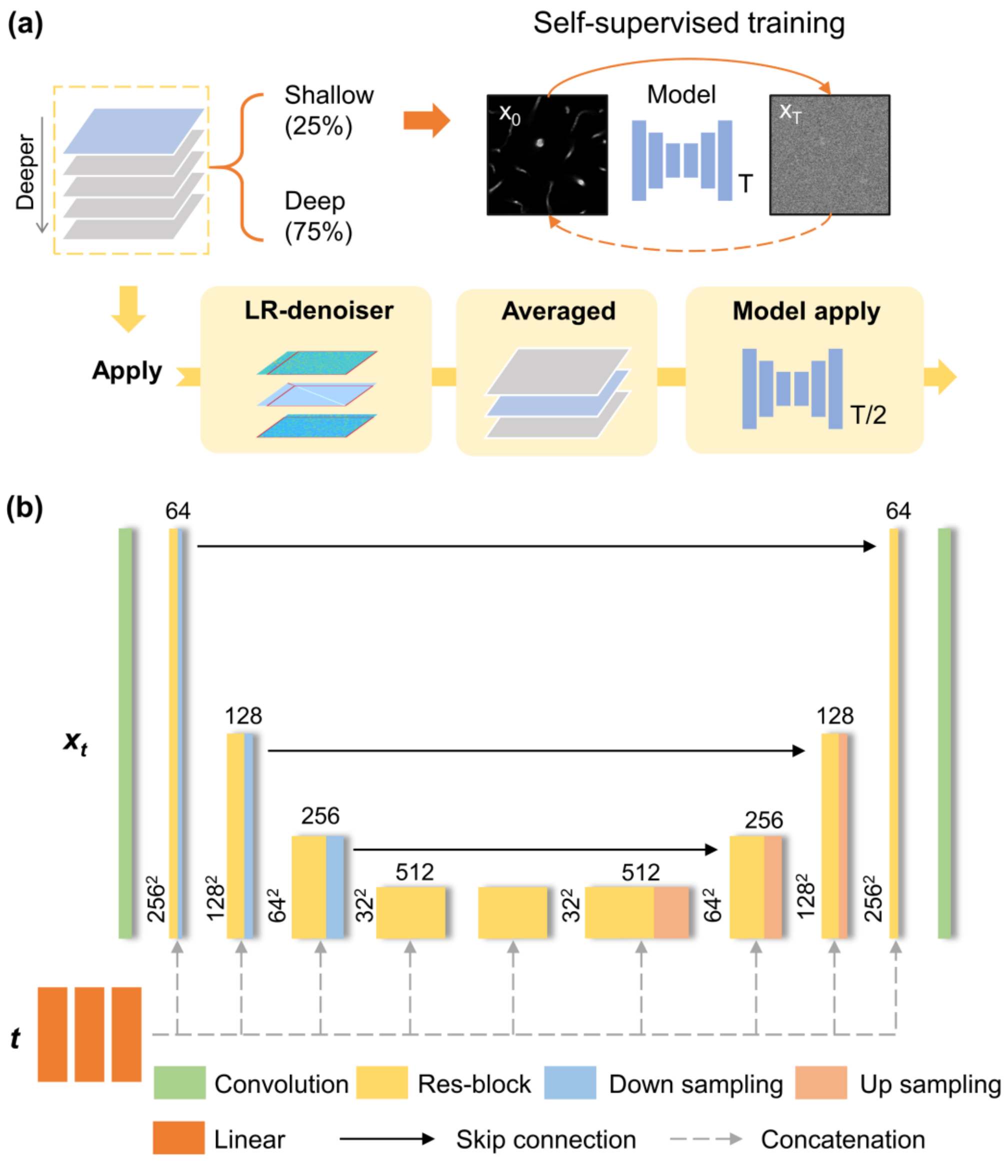

Fig. S1 Overview of the LRDM method. (a) Workflow of the LRDM training and the procedure of LRDM usage. (b) Architecture of the model framework, which takes the noised image (x_t) and the noise step (t) as inputs and yields a denoised image as the output.

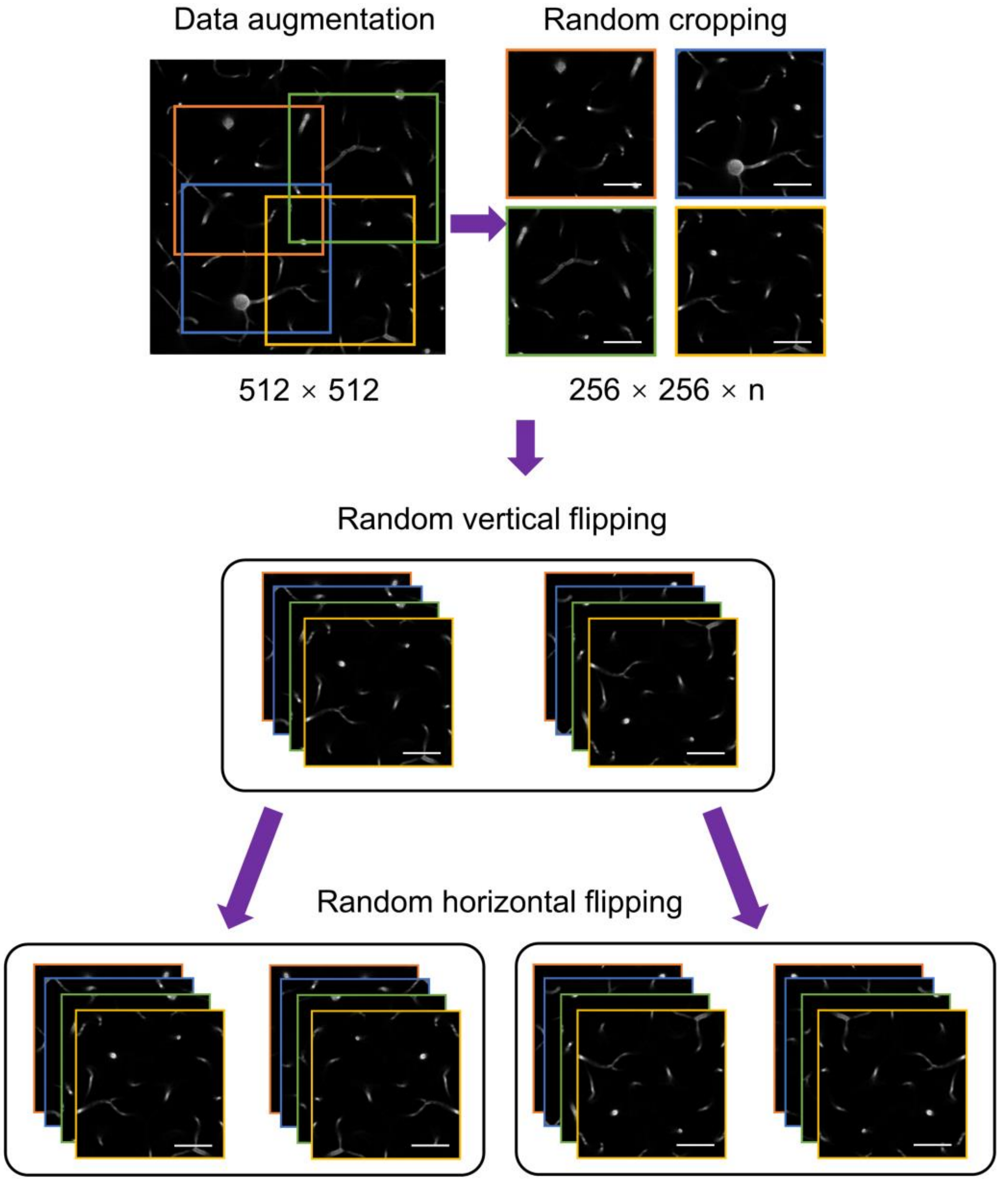

Fig. S2 Data augmentation strategy. Shallow images are considered as ground truth for training. In each iteration, a 256 × 256 sub-image is randomly cropped from a 512 × 512 raw image, and then undergoes random vertical and horizontal flipping to augment the training data pool.

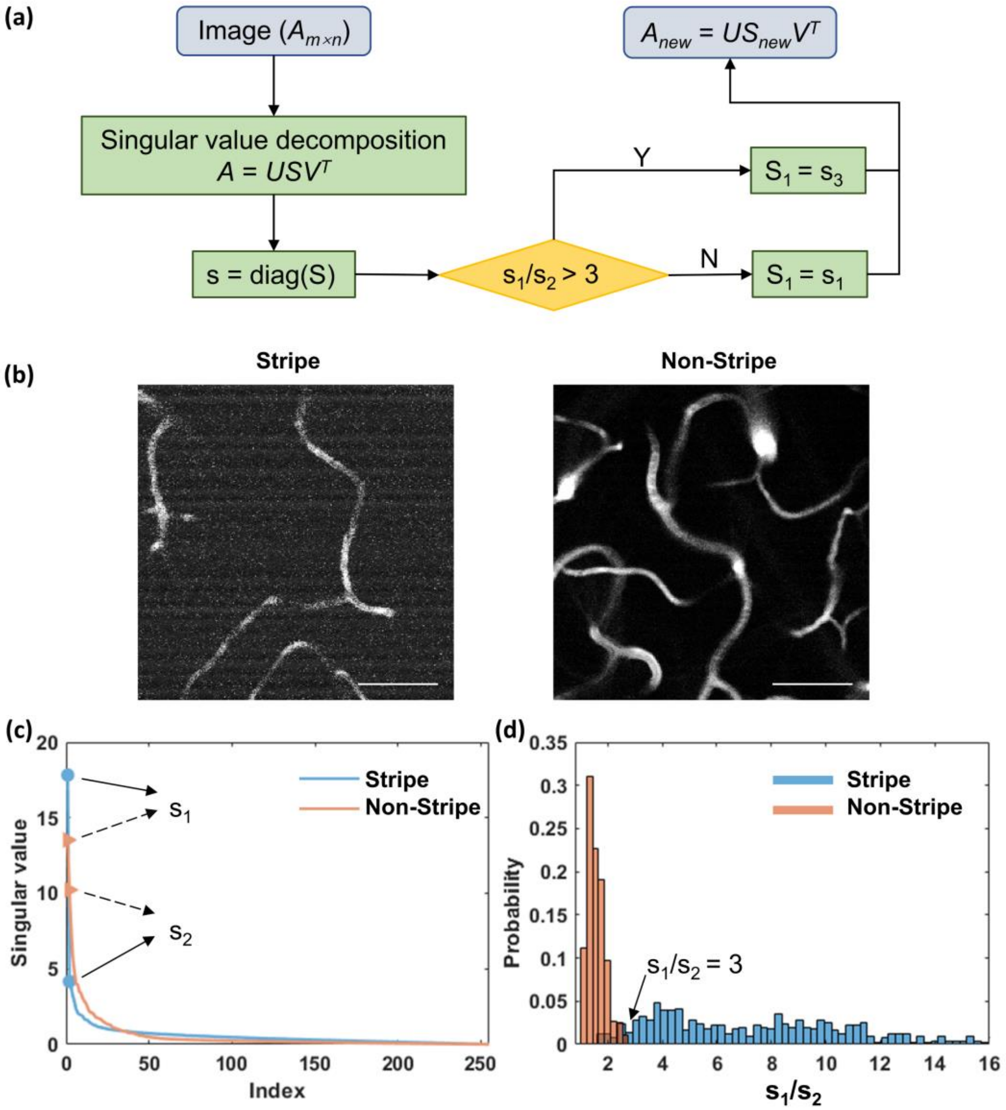

Fig. S3 Overview of the LR-denoiser workflow. (a) Flowchart of the LR-denoiser. A singular value matrix ***S*** along with its diagonal vector ***s*** are obtained after performing the singular value decomposition (SVD) on the image matrix ***A***. To compare the first value (s_1) with the second one (s_2) in the vector ***s***, we perform the following steps: if $s_1/ s_2 > 3$, then replace the first singular value (S_1) in the singular value matrix ***S*** with s_3; otherwise, leave it unchanged. (b) Examples of the images defined as ones of stripe or non-stripe. Scale bar, 50 µm. (c) The plots of corresponding singular values from representative images shown in (b). (d) Histogram distributions of s_1/ s_2 singular value ratios of images with/without stripe (n = 500).

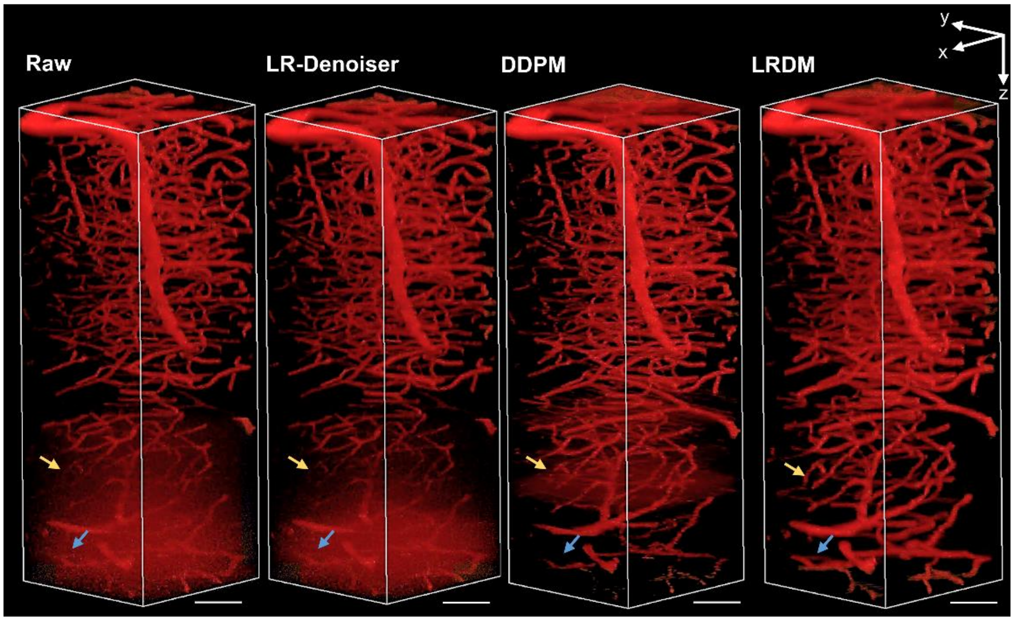

Fig. S4 3D reconstructions of raw images and images dealt with LR-denoiser, DDPM and LRDM. Scale bar, 100 μm.

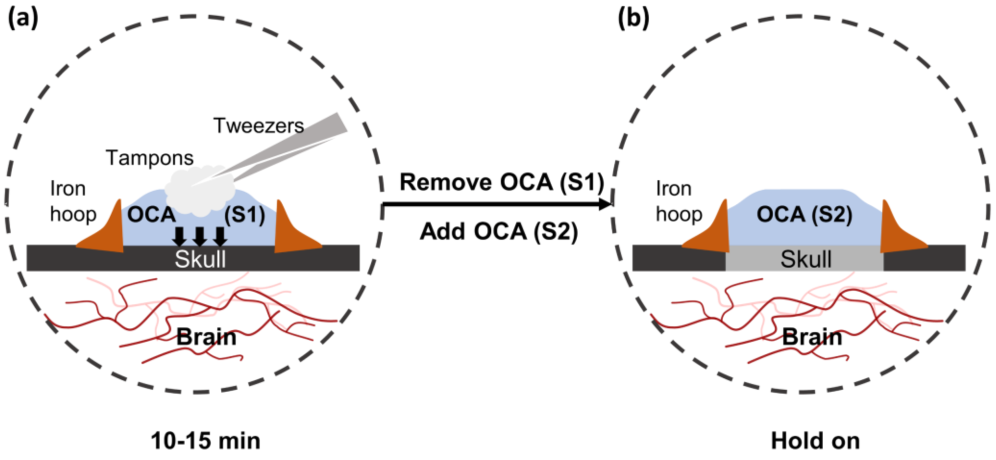

Fig. S5 Schematic of skull optical clearing procedure. (a) The skull is dealt with a saturated supernatant solution of urea and ethanol (termed S1) containing optical clearing agents for 10-15 min. Tampons are applied to press S1 to seep it down into the skull. (b) A high-concentration sodium dodecyl benzenesulfonate (termed S2) containing optical clearing agents was added to replace S1 for the subsequent 3PM imaging.

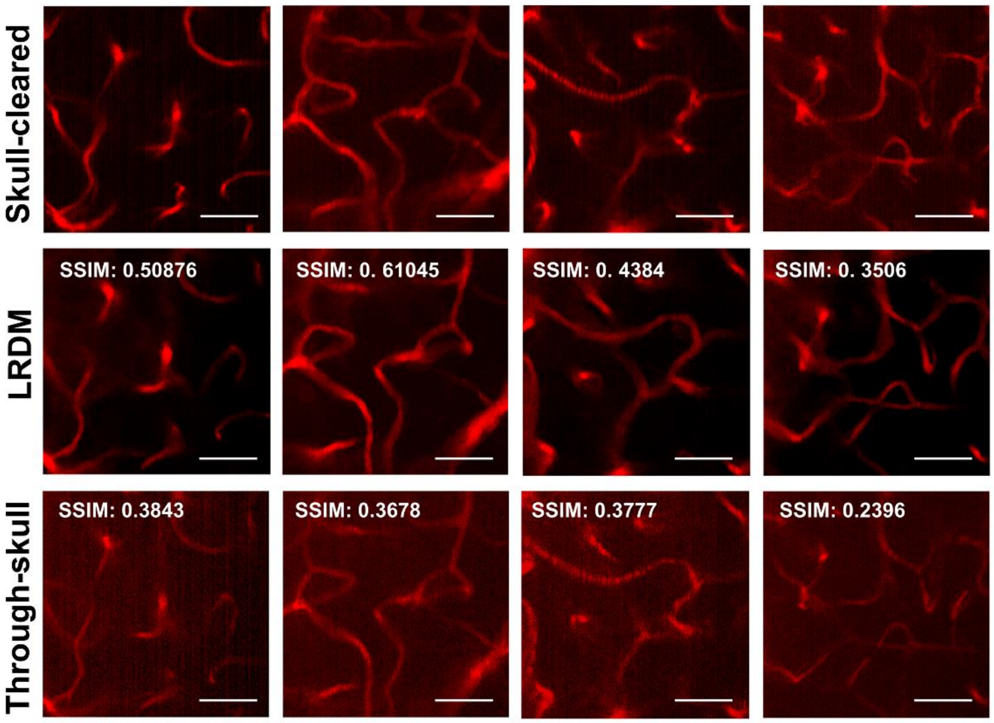

Fig. S6 Comparison of representative images, including skull-cleared images (skull-cleared, top), LRDM-enhanced images (LRDM, middle) and through-skull images (through-skull, bottom). Scale bar, 50 μm.

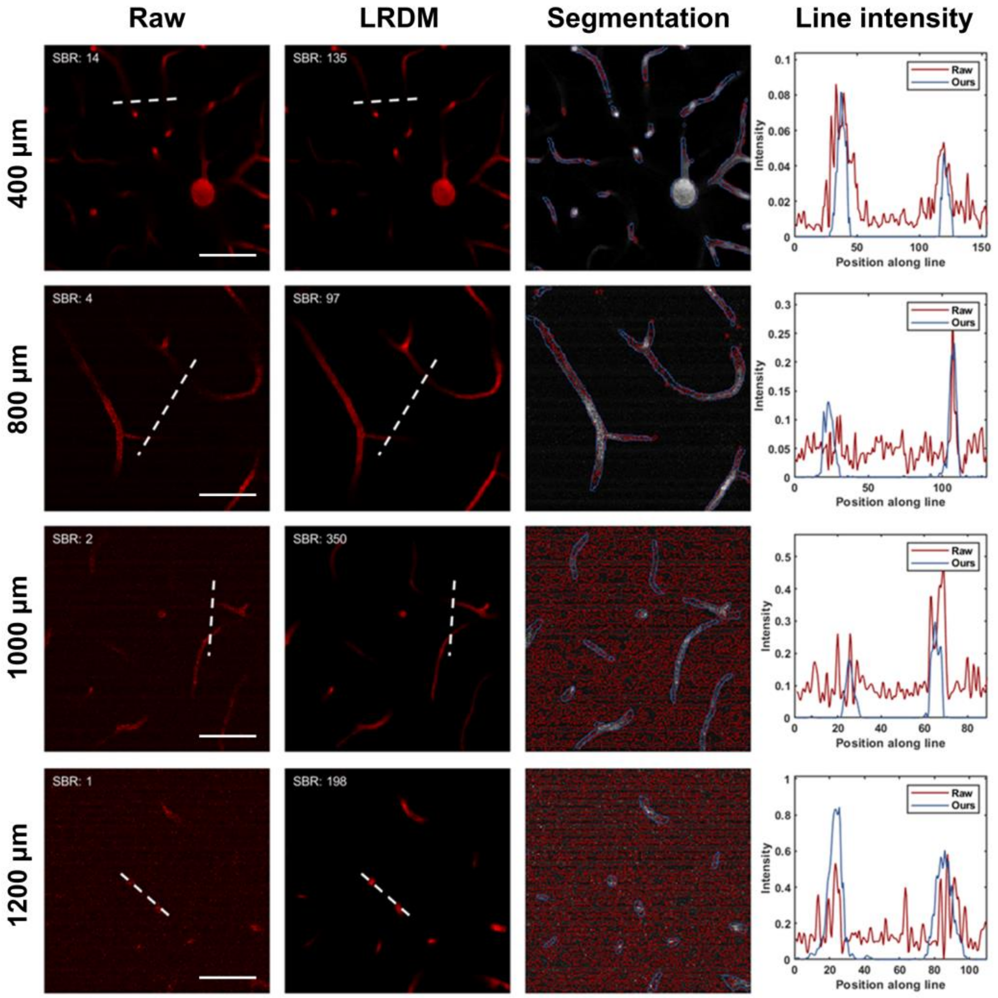

Fig. S7 Representative images and vessel segmentation results at different depths before and after LRDM enhancement (left), and intensity profiles along the white dashed lines (right). Scale bar, 50 μm.

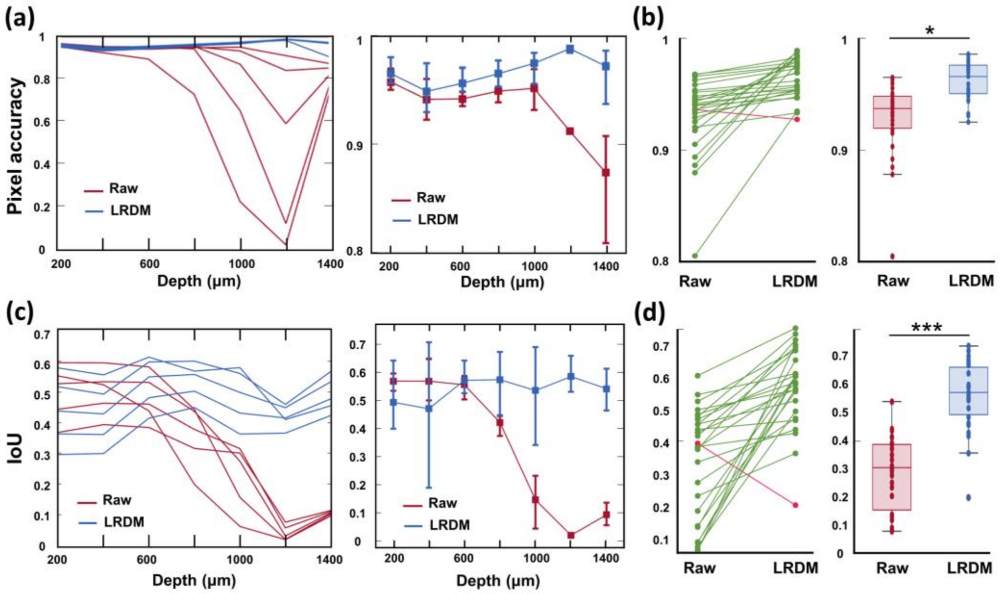

Fig. S8 Segmentation accuracy results calculated by different evaluation functions. (a) Pixel accuracy as a function of imaging depth, with the proportion-adjusted Otsu thresholding (left) and the weighted optimal thresholding (right). (b) Slope chart (left) and box plots (right) of pixel accuracy. (c) Intersection over Union (IoU) as a function of imaging depth, with the proportion-adjusted Otsu thresholding (left) and the weighted optimal thresholding (right). IoU is a performance metric used to evaluate the accuracy of annotation, segmentation, and object detection algorithms. (d) Slope chart (left) and box plots (right) of IoU. $n = 27$ images from 4 mice. *, $p < 0.05$; ***, $p < 0.001$.

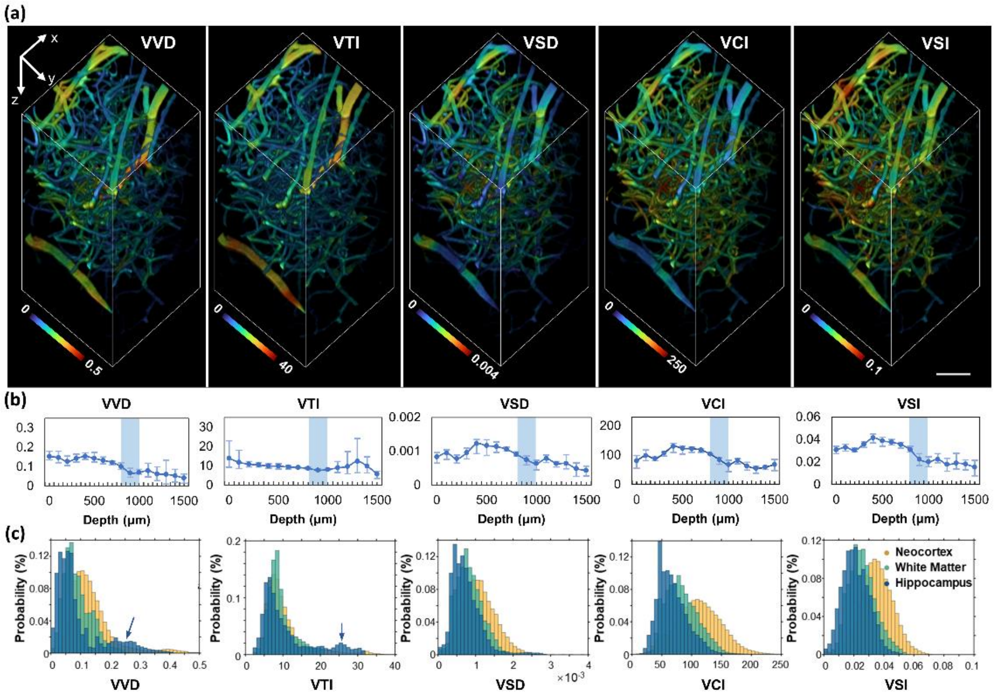

Fig. S9 Results of multi-parametric analysis. (a) 3D reconstruction of color-coded maps by different morpho-structural metrics. Scale bar, 100 μm. (b) Corresponding depth-dependent profiles. The blue shadow corresponds to the white matter region. (c) Corresponding distribution histograms.

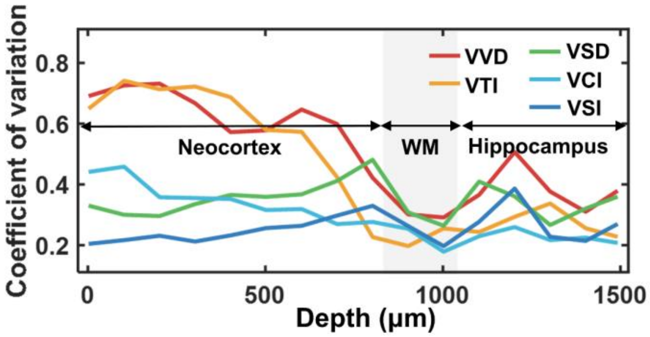

Fig. S10 Depth-dependent profiles of coefficient of variation (CV) for different morpho-structural metrics.

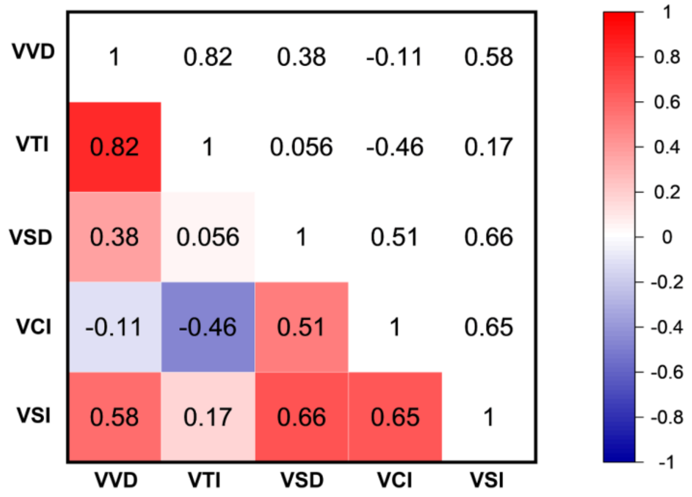

Fig. S11 Correlation heat map among metrics of VVD, VTI, VSD, VCI and VSI.

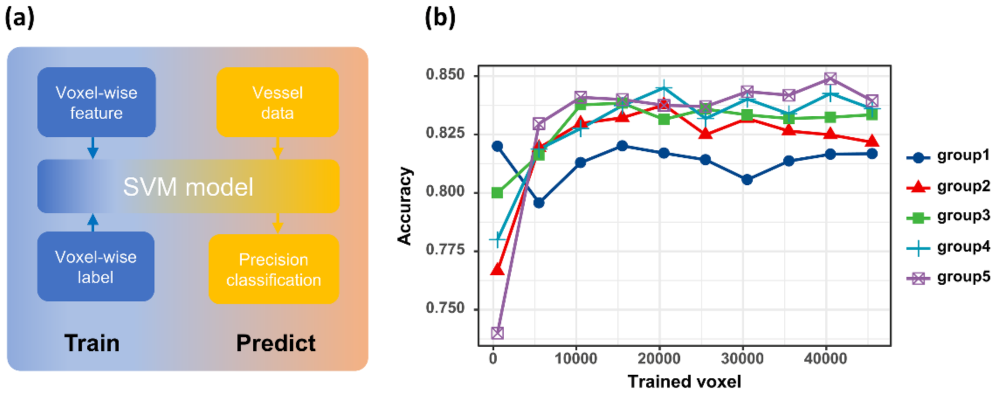

Fig. S12 Model training for recognition of brain regions based on vascular morphology. (a) Model training flow chart based on SVM. (b) The classification accuracy as a function of number of trained voxels obtained using different combinations of metrics. Group 1: raw image intensity. Group 2: VVD only. Group 3: VVD + VSD + VCI. Group 4: VVD + VTI + VSD + VCI. Group 5: combination of all the metrics.
